# A daily global mesoscale ocean eddy dataset from satellite altimetry

**DOI:** 10.1038/sdata.2015.28

**Published:** 2015-06-09

**Authors:** James H. Faghmous, Ivy Frenger, Yuanshun Yao, Robert Warmka, Aron Lindell, Vipin Kumar

**Affiliations:** 1 Department of Computer Science, The University of Minnesota—Twin Cities, Minneapolis, MN, USA; 2 Department of Atmospheric and Oceanic Sciences, Princeton University, Princeton, NJ, USA; 3 Department of Population Health Science and Policy, Icahn School of Medicine at Mount Sinai, New York, NY, USA

**Keywords:** Physical oceanography, Climate and Earth system modelling

## Abstract

Mesoscale ocean eddies are ubiquitous coherent rotating structures of water with radial scales on the order of 100 kilometers. Eddies play a key role in the transport and mixing of momentum and tracers across the World Ocean. We present a global daily mesoscale ocean eddy dataset that contains ~45 million mesoscale features and 3.3 million eddy trajectories that persist at least two days as identified in the AVISO dataset over a period of 1993–2014. This dataset, along with the open-source eddy identification software, extract eddies with any parameters (minimum size, lifetime, etc.), to study global eddy properties and dynamics, and to empirically estimate the impact eddies have on mass or heat transport. Furthermore, our open-source software may be used to identify mesoscale features in model simulations and compare them to observed features. Finally, this dataset can be used to study the interaction between mesoscale ocean eddies and other components of the Earth System.

## Background & Summary

Mesoscale ocean eddies (eddies) are coherent rotating vortices of water with radial scales ranging from 25–250 km and lifetimes of 10 to 100 days^1^. Eddies play a significant role in the mixing and transport of heat, salt, and biogeochemical tracers across the global oceans. Moreover, eddies have been shown to influence near-surface winds, clouds, and rainfall within their vicinity^2,3^ as well as marine ecosystems^4–6^. Finally, eddies may act as a moderating factor in global climate change^7^. Thus, understanding global ocean eddy dynamics and their role in influencing various oceanographic, atmospheric, and biological phenomena is of keen scientific interest.

We present the *OpenEddy* data which consist of global daily eddy features, trajectories, and source code software to monitor mesoscale ocean eddy activity in global sea level anomaly (SLA) data. Unlike other data, the eddy features are not filtered based on any particular criterion, except for a minimal size of 4 pixels. Depending on the application, researchers may further filter the data based on their needs (size, amplitude, deformation, lifetime, *etc*.) The open-source software also allows researchers to collect eddy data based on any set of parameters in a parameter-free fashion and is computationally efficient. Finally, we provide daily eddy trajectories that can be downloaded and analyzed immediately as the largest publicly available daily eddy dataset.

Eddy detection based on SLA became feasible in the early 1990s with the concurrence of two satellite altimetry missions. A merged product of (at least) two missions provided high enough data resolution to enable the detection of mesoscale eddies. Over the past ten years, the number of studies involving automated eddy detection in observational and model data in both regional and global studies has grown tremendously^2,3,5,6,8–28^. While eddy detection research has been very active, only a few studies make public their eddy identification software or trajectories.

For identification software, we are aware of four projects^29–32^. However Lily *et al.*’s^29^, method focus on extracting oscillatory features in Lagrangian trajectories, such as from surface drifters^30^ and Petersen *et al.*^31^ point out that their eddy detection software’s performance diminishes significantly when analyzing global data. Nencioli *et al.*’s^32^ highly parametrized method based on the ocean flow structure requires careful tuning and a sufficiently resolved flow field. Finally, although these projects provide their source code for download, such software is not hosted in a repository.

For datasets of eddy trajectories, to our knowledge, only Chelton *et al.*(CSS11)^33^ publicly provide global eddy trajectories. There are several differences between our data and CSS11. First, our data are based on the most recent version of postprocessed daily SLA estimates, while the version used by CSS11 consisted of weekly estimates. In addition, this new SLA product better resolves mesoscale features (see AVISO webpage, http://www.aviso.altimetry.fr, and the User Handbook). Second, the trajectories reported by CSS11 are for a single set of parameters such as 1 cm amplitude and a 4-week minimum lifespan. Third, CSS11 reported two limitations of their identification and tracking procedures which make their methods prone to merging nearby eddies and prematurely terminating trajectories (see Appendix B in CSS11^33^)—issues we resolve in our eddy identification and tracking procedures. Finally, of the features reported by CSS11, only an estimate of the eddy’s diameter without the exact contours of the eddies are given (i.e. grid cells that are part of the feature). We provide the exact eddy contour as it is critical for studying eddy impacts on other phenomena. A summary of these differences is given in Table 1(available online only).

The automatic identification of eddies in global SLA data comes with notable uncertainties due to the lack of consensus about the spatio-temporal signatures eddies leave in the SLA field as well as how well do existing satellite products resolve such a signal. Furthermore, merged satellite products such as the one used in this study are susceptible to noise and may carry artificial signals. To reduce the chance of false discoveries, most studies impose strict quality thresholds on the features identified, such as minimal lifespan or size. Although filtered datasets are meant as a precaution to only include the most robust features, any strict threshold on eddy size, amplitude, or track length will indiscriminately remove both spurious and real features. This is especially the case since the physical characteristics of a robust eddy are highly variable in space and time^34,35^. Our software identifies eddies with any set of parameters, which is particularly valuable since the appropriate threshold choice varies across regions and studies. Moreover, this parameter-free approach enables testing results for sensitivities to threshold parameters.

There are two major uncertainties associated with our eddy identification algorithm, both stemming from our operational definition of an eddy. We define eddies as the outermost closed-contour SLA containing a single extreme (maximum/minimum). First, using a geometric eddy definition as opposed to a physical one, introduced some uncertainty as the eddy boundary is not necessarily associated with a physical property of the eddy. Second, as we constrain features to have both a single extremum, small features that are in close proximity might cancel each other out and not be detected. Despite these uncertainties, our algorithm recovers 96.4% of features as identified by domain experts.

Our data and software have significant reuse value. They enable researchers to directly analyze global mesoscale eddy properties and dynamics, without the need to implement an eddy identification method. Further, eddy trajectories can be complemented with additional satellite-based observations or *in-situ* measurements to estimate the impact of eddies on tracers and to gain a better understanding of the interactions between eddies and other features of the Earth System such as marine predators. In addition to scientific applications, a comprehensive set of eddy features that are not filtered based on expert criteria allows researchers to develop novel feature tracking methods. Finally, the data and methods can be used to validate AVISO data by comparing features identified in post-processed and along-track AVISO products.

## Methods

### Brief review of previous work

Global ocean eddy monitoring has become possible thanks to Earth-orbiting satellite data. Given the ubiquity of eddies, autonomous methods were needed to extract mesoscale features with limited human input. The omnipresence of ocean eddies became apparent from early satellite data of ocean color and sea surface temperature in the late 1970s^36,37^, and those variables have been used for eddy identification since then^38–40^.

The advent of sea level anomaly observations from satellite radar altimeters provided researchers with data that are more intimately related to ocean eddy activity than the previous datasets. Eddies are classified based on their rotational direction as either cyclonic if they rotate counter-clockwise (in the Northern Hemisphere) or anti-cyclonic otherwise. Cyclonic eddies cause a decrease in SLA and elevations in subsurface density surfaces. Anti-cyclonic eddies, cause an increase in SLA and depressions in subsurface density surfaces. These characteristics enable the identification of ocean eddies in SLA satellite data, where anticyclonic eddies manifest in the form of closed-contour positive SLA, while cyclonic eddies are reflected in closed-contour negative SLA. The earliest automated eddy detection methods in SLA data were based on physical criteria and relied on a measure of rotation and deformation in fluid flow known as the Okubo-Weiss (W) parameter^41,42^. In such studies, eddies were defined as features where the W-parameter was below an expert-specified negative threshold. The W-parameter typically was applied regionally with a region-specific threshold^43,44^. Also the first global observational eddy monitoring study was based on the W-parameter^45^, using a globally uniform threshold. Petersen *et al.* carried out a global modeling study where the W-parameter was used with additional constraints on the shape of the eddies^31^.

However, the W-parameter method has been criticized for its dependence on thresholds as well as its sensitivity to noise^33,46,47^. As a result, later research efforts favored new methods, for instance, wavelet analysis^48,49^, winding angle^50,51^, reversal of the flow field^32^, the derivation of the flow field from observed sea surface temperatures (SST)^40^ and subsequently applying Nencioli *et al.*^32^ method, outermost closed SLA anomaly contours^30,33,52^, or a combination of physical and geometric methods^53,54^. While these approaches attempt to alleviate the threshold-dependent nature of the W-method, they too employ expert-defined parameters to determine what constitutes an eddy. For example, works from Fang and Morrow^55^ and Chaigneau and Pizarro^56^ considered eddies that only pass the threshold value of ±10 cm and ±6 cm respectively. While Faghmous *et al.*^52^  imposed a minimal convexity condition to ensure that only the most compact features were preserved.

A growing development is a computationally laborious aims at detecting (sub)mesoscale transport-barriers and ‘Lagrangian coherent structures’, with a focus on vortices that trap fluid and materials^46,57–63^. The present study focuses on an SLA-based method that detects eddies independent of their trapping ability.

**Figure 1:**
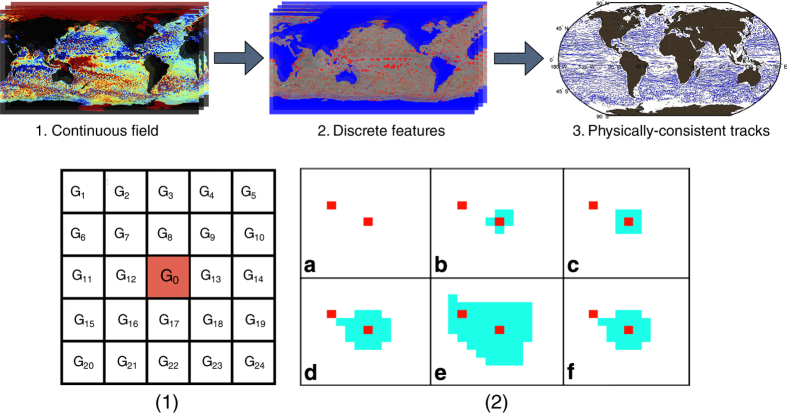
High-level overview of eddy identification schemes. Top panel: a common process most autonomous eddy identification schemes follow. Starting from a continuous SLA field (left panel), discrete features are isolated from the background (red features in middle panel). Each satellite snapshot is analyzed independently, then the features from each snapshot are associated with nearby features in space and time to produce physically consistent eddy trajectories (right panel). Given the large number of features identified at each time-step, many features are discarded if they fail certain expert-defined criteria such as minimal size or minimal trajectory duration. Bottom panel: A demonstration how our proposed method identifies eddies (1) An illustration how we determine if a grid cell is an extremum. For each grid cell *G*_0_ we compare its SLA value to its 24 neighbors in a 5×5 neighborhood. *G*_0_ is labeled as an extremum if: SLA(*G*_0_)<SLA(*G*_*i*_) or SLA(*G*_0_)>SLA(*G*_*i*_); i=1:24. (2) The six steps that lead to identifying a cyclonic eddy. (**a**) All minima within the sample region are identified and highlighted in red. (**b**–**d**) Starting from the minimum at the center of the panel, iteratively threshold the SLA to find a closed-contour SLA that is greater than the minima. The eddy’s extent is highlighted in light blue. (**e**) The eddy’s extent engulfs two minima and the thresholding is terminated; set the feature’s contour as that of the previous step. (F) The final contour of the eddy.

While the exact techniques may vary, SLA-based methods follow a similar work-flow that is summarized in Fig. 1. One should note that various eddy detection approaches may also employ different pre- and post-processing steps. Starting from a single satellite snapshot of SLA, these methods isolate the sea surface height anomalies that could potentially be eddies. However, methods that extract eddies from an SLA background are error prone because they attempt to infer the presence of a three-dimensional phenomenon using only surface information that is noisy and impacted by processes other than eddies. As a result, the discrete items extracted from the continuous field are filtered as a precautionary measure to exclude any uncertain features. The criteria that make a feature uncertain vary from study to study but generally involve sg automated eddy detection trict expert-defined criteria such as minimal size or shape; and features that fail such criteria are removed from the analysis. This process is then repeated for every available satellite snapshot. Finally, eddy trajectories are constructed by associating features in one time-step to the nearest and most similar feature in space and time. The tracking phase also includes expert-defined criteria such as predefined search windows in space and time. Finally, some studies filter final results to only include eddies that persist for several weeks or months.

Our global ocean eddy monitoring technique comprises two steps. First, eddy-like features are identified as closed-contour SLA that have a single extremum in each satellite snapshot. Once all features have been identified, we track each feature in time by associating a feature in one time-step to the nearest feature in the subsequent one.

### Autonomous eddy identification

The autonomous eddy identification scheme^64^ starts from the simple notion that every eddy should have a single extremum. An extremum is defined as a grid cell whose SLA is greater (maximum) or less (minimum) than its 24 neighbors in a 5×5 neighborhood. This is achieved by visiting every grid cell and comparing its SLA value to the grid cells that are up to 2 cells to the left, right, top, bottom, and diagonal for a total of 24 comparisons (see Fig. 1 Bottom panel 1). It is possible to define extrema over a 3×3 or 7×7 neighborhood but we found a nine-pixel neighborhood to result in too many extrema in close proximity, since it is more likely to be an extremum in a nine-pixel neighborhood by random chance. A 7×7 neighborhood (49 pixels) was too strict and would miss small eddies.

Thus, starting from all possible extrema in a given SLA snapshot, we select the features that have one of the available extrema while also being a closed-contour SLA. Cyclonic eddies have a single minimum surrounded by a closed-contour SLA. While anticyclonic eddies would have a single maximum surrounded by a closed-contour SLA. This simple observation, that each eddy ought to contain a single extremum, allows us to address CSS11’s limitation of artificially merging nearby eddies^30,33,52,64^. A video showing how our method avoids merging nearby eddies is available at: https://vimeo.com/108387905.

To determine the exact contour of the eddy, starting from an extremum, the algorithm searches for the largest possible contour that would allow the feature to not violate the assumption that an eddy can only have a single extremum within its interior. The eddy’s core is constructed by iteratively thresholding the neighborhood around the extremum and assigning a binary value of zero or one depending on whether the SLA at a grid cell exceeded a certain threshold or not. The threshold is initially set to the extremum’s value and is gradually increased (decreased) by a user-defined step. As the threshold increases (decreases) the contour of the feature grows until another extremum is included within its interior. At that point the contour of the eddy is decreased to the previous threshold when it still contained a single extremum. This unconstrained approach to determining the eddy contour may sometimes lead to overestimating the eddy’s size. However, based on visual verification this does not seem to be a major issue.

Figure 1 (bottom panel 2) shows how the algorithm works for a cyclonic feature. The algorithm identifies cyclonic and anticyclonic eddies separately. For cyclonic eddies, the algorithm examines every minimum identified in a single SLA snapshot. For a given minimum, starting at that SLA value, a threshold value is gradually increased in 0.05 cm increments. CSS11 (ref. 33) and others use a 1 cm step, but we found that a finer threshold step leads to more accurate eddy sizes and amplitudes. This is because once the algorithm extends the eddy contour to contain two extrema, it would reduce the size of the eddy by the value of the thresholding step since it must revert back to the threshold value when the eddy contained a single extremum. Hence, the larger the threshold step the bigger the step back, which could result in under-estimating some eddy properties. The threshold step size does affect the algorithm’s computational run-time, however our parallelizable software allows to use fine threshold steps with reasonable compute time.

Grid cells that have SLA anomalies greater than the current threshold are set to one, while those below the threshold are set to zero. All adjacent grid cells that have a value of one are considered a connected component that form the eddy’s body. As the threshold continues to increase, more grid cells are added to the connected component. The thresholding stops if any of the newly added grid cells is labeled as an extremum. The process is then repeated for anticyclonic eddies, where the algorithm starts at a local maximum and gradually decreases the threshold by 0.05 cm. All values that are below the threshold are set to one while the others are set to zero. A video that shows how eddies are identified on a larger scale in a single SLA snapshot can be viewed at https://vimeo.com/108387776.

This eddy identification approach is considered ‘parameter-free’ because the stopping conditions of the iterative thresholding process are solely dependent on the presence of other extrema within an eddy’s neighborhood. This allows for the removal of most of the expert parameters imposed by other methods such as maximum size or minimum amplitude. While expert-defined parameters are common, imposing a strict and arbitrary threshold will certainly cause numerous eddies to be discarded, especially given the large spatial variability in eddy properties. A parameter-free approach however, keeps all features identified as long as they are 4 grid cells or larger. While such an approach would result in spurious features, researchers are then free to remove any features that are not pertinent to their analyses.

### Automatic eddy tracking scheme

Once all eddy-like features are identified in each SLA snapshot, the following eddy tracking procedure is applied and was adapted from^33^: For each eddy feature identified at time *t*, the features at time *t*+1 are searched to find the closest feature within a pre-defined search space based on the theoretical distance an eddy can travel during the period between two successive time frames. To constrain the search space, we use an estimate of an eddy’s expected propagation speed—the phase speed of long (nondispersive) baroclinic Rossby waves, calculated from the Rossby radius of deformation as presented in Chelton *et al.*^65^. Other factors may impact propagation distance, however, the measure used has been found to sufficiently agree with the eddy propagation speed^45^. In the event that a feature at time *t*+1 is equidistant from two features, it is assigned to the first encountered feature.

Once a feature at time *t* is associated with another feature at time *t*+1 their size is compared to ensure that they are reasonably similar from a physical standpoint. In order for a feature to be associated with another in the subsequent time frame, both the size and amplitude in *t*+1 must be within 0.25 to 2.75 times that of *t*.

Due to the noise and sampling errors in the AVISO product, eddies may temporarily ‘disappear’ and reappear a few time-steps later^33^. An automatic tracking procedure that does not account for this bias would prematurely terminate tracks. To address this challenge, we extend the method proposed by CSS11 by allowing features to be unassociated for a user-defined number of time-steps before terminating the track. This was a necessary improvement as tracks were being prematurely terminated at a higher rate in the daily SLA data compared to the rates reported by CSS11 in the weekly SLA field (based on visual inspections of the trajectories).

**Figure 2:**
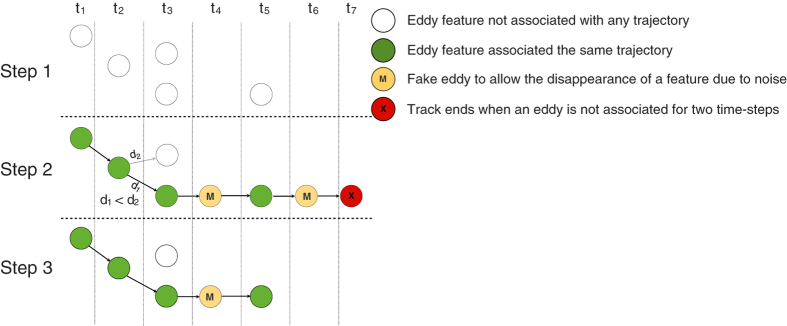
An illustrative example of how the eddy tracking algorithm works. The horizontal panels represent 7 time steps (*t*_1_ to *t*_7_). The vertical panels highlight three distinct steps of the algorithm. Step 1: all eddy features are identified in the seven consecutive time-steps. At this stage no eddy trajectories exist. Step 2: For each time step *t*_*i*_, associate each eddy from *t*_*i*_ to *t*_*i*+1_. In this case, the lone eddy from *t*_1_ is associated with the lone eddy in *t*_2_. At *t*_2_ there is choice to be made between the two candidate eddies in *t*_3_, both of which are within the eddy’s search space. The eddy is associated with the nearest eddy. At *t*_3_ there are no candidate eddies to associate with in *t*_4_, however, the track is not terminated as eddies might ‘disappear’ due to noise. In the event of a missing eddy, a fake eddy is inserted with an ‘M’ label to differentiate it from real eddies. The process is continued until two consecutive ‘M’ eddies are observed in which case a termination eddy denoted by ‘X’ is inserted. Step 3: The bottom panel shows the resulting trajectory with one feature marked as ‘M’ which signifies a ‘fake’ eddy (which should be discarded when studying dynamics, *etc.*).

In the event that a feature is left unassociated at time *t* we simply insert a temporary ‘fake eddy’ for that time step. The fake eddy is a copy of the last observed eddy in terms of properties (size, amplitude etc) and is positioned along the same propagation axis of the current trajectory using the propagation speed observed in the most recent time-step for that track. The algorithm subsequently searches for a feature to associate the ‘fake eddy’ to in the next time frame. If an eddy is found, the position of the ‘fake eddy’ is re-interpolated as the average position between the last real eddy in the track and the newly associated eddy. The fake eddy is then flagged for bookkeeping purposes to not be included in any analyses (see Fig. 2). If an eddy is not found in the next time-step, the algorithm will place additional temporary ‘fake eddies’ until the number of user-defined ‘misses’ is exceeded, at which point the string of ‘fake’ eddies at the end of the track are removed and the track is terminated. It should be noted that the uncertainty and likelihood of errors in associating eddies to the correct track increase with the number of permissible unassociated time-steps. We have used only a single day as the tolerance level for trajectories to be extended in our data. A video showing global eddy trajectories in weekly SLA data can be found at https://vimeo.com/108272705.

Similar to eddy identification, there are several variations of the tracking algorithm, some of which involve numerous parameters^47,66^. Furthermore, the maximum propagation distance to constrain the search space can be modified to include uncertainty or geographic variability such as advection by the mean flow. Finally, we have found that a more straightforward and computationally efficient approach to address the ‘disappearing’ eddies is by post-processing the data. The post-processing approach examines every terminated trajectory and searches in the neighborhood if another trajectory started a day after the current trajectory ended. However, a post-processing approach would work well only for daily data as eddies propagate for relatively longer distances on weekly time-scales which increases the likelihood of mistakenly linking two different trajectories.

### Experimental setup

These are the steps taken to produce our data [Data Citation 1]. The altimeter products were produced by Ssalto/Duacs and distributed by the Archiving, Validation, and Interpretation of Satellite Oceanographic (AVISO), with support from CNES (http://www.aviso.altimetry.fr/duacs/). The data were ‘Delayed Time’ ‘all sat merged’ global daily mean sea level anomalies on a 0.25° grid from January 1993 through May 2014 (http://www.aviso.altimetry.fr/es/data/products/sea-surface-height-products/global/msla.html#c5122). The ‘all sat merged’ data consist of datasets with up to four satellites at a given time, using all missions available at a given time. Sampling and long wavelength errors determination are thus improved, so this series is better in quality but not homogeneous over the entire time span of the study. In the Duacs 2014 version (v15.0), the reference period of the Sea Level Anomalies is based on a 20-year period of 1993 to 2012.

### Code availability

We provide open-source software to identify and track ocean eddies from SLA data^67^. The algorithm was run on the SLA anomaly data described above without any filtering or preprocessing steps. The source-code for the eddy identification algorithm is implemented in *scan_single.m* in the source-code repository available on the Zenodo repository^67^. The source code in^67^ is a stable version as of December 2014. We also provide a continuously-updated version at https://github.com/jfaghm/OceanEddies. The identification procedure was executed with the following parameters:

cycEddies=scan_single(SLA, lat, lon, 19930101, ‘cyclonic’, ‘v2’, areaMap);

antEddies=scan_single(SLA, lat, lon, 19930101, ‘anticyc’, ‘v2’, areaMap); where SLA is the sea surface height data, lat and lon are the latitude and longitude vectors corresponding to the 720×1440 grid of SLA data (provided by AVISO), 19930101 is an example date for a particular data file, ‘cyclonic’ or ‘anticyc’ is the type of eddies desired, ‘v2’ is the version of the algorithm to use, and areaMap is an array containing the surface area in squared kilometers of each grid cell (needed to compute eddy surface area).

Once all features were identified, eddy trajectories were prodcued using *tolerance_track_lnn.m* (LNN stands for ‘local nearest neighbor’). We allowed for an eddy to be unassociated for a single time-step. If the eddy is unassociated for two consecutive time-steps the track was terminated.

track_lnn example: cycTracks=track_lnn(eddies_directory, eddy_rotation, temporal_frequency). The first parameter is the path to the directory where the eddies were saved. The second parameter is the type of eddy (‘cyclonic’ or ‘anticyclonic’). The third parameter is the temporal resolution of the data (1 for daily or 7 for weekly).

tolerance_track_lnn example: cycTracks=tolerance_track_lnn(eddies_directory, eddy_rotation, temporal_frequency, tolerance, minimumArea). The first parameter is the path to the directory where the eddies were saved. The second parameter is the type of eddy (‘cyclonic’ or ‘anticyclonic’). The third parameter is the temporal resolution of the data (1 for daily or 7 for weekly). The fourth parameter is the number of time-steps that the algorithm will tolerate an eddy to go undetected for, and the fifth parameter is a minimum area criteria that an eddy has to meet to be included in the track. This minimum area parameter is added so that small eddies can be filtered out before being tracked. All eddies are preserved, but only eddies that meet the minimum area criteria will be eligible for being tracked.

## Data Records

Our data [Data Citation 1] constitute of two components. First, we provide all mesoscale eddy features that are at least four grid cells large. These features represent the closed contour positive and negative SLAs identified by our eddy identification algorithm, *without* tracking. Researchers can then filter the data based on their needs (e.g., 10 grid cell minimum size) and apply our eddy tracking algorithm to produce eddy trajectories. Second, we provide one set of trajectories for eddies with sizes of at least nine grid cells and 1 cm amplitude. These trajectories are stored in a tracks file with references to access the eddy properties (size, amplitude, etc.) from the original dataset. These trajectories are only an example and users should feel free to construct trajectories based on their own needs.

### Eddy features

For each daily SLA snapshot, we identify approximately 2,700 cyclonic and 2,700 anticyclonic eddies. Different studies have different quality metrics on what constitutes an eddy, and by providing all possible features, researchers are still able to apply their own quality metrics to the data. For each day of the daily data, we have two files saved: one for all the cyclonic and the other for the anticyclonic features identified. A sample data record that includes all the information saved per eddy snapshot is listed in Supplementary Table 1.

### Eddy trajectories

The trajectory data are formatted in a table where each row is a time-step with the date, latitude, longitude, and eddy identifier. Collectively the trajectories are stored in a *cell array* or an array of tables where each cell contains a table that details the entire trajectory of a single eddy. Table 2 (available online only) shows a sample eddy trajectory file that contain the following fields:

1. *Track ID:* the trajectory’s unique ID. All observations within the same trajectory would have the same ID.
2. *Latitude:* the latitude of the eddy’s centroid in degrees North.
3. *Longitude:* the longitude of the eddy’s centroid in degrees East.
4. *Time ID:* the index of the date at which the eddy was observed (ranges between 1 and 7746 for daily data)
5. *Eddy index:* the eddy’s unique index within that date’s eddy file saved in the eddy identification step described previously.
6. *Flag*: an integer denoting whether the feature is a real or a ‘fake’ eddy from a missing observation. 0 means real and −1/1 means ‘fake’ for cyclonic and anticyclonic eddies respectively.
7. *Cyc*: The rotational direction of the eddy. −1 for cyclonic and 1 for anticyclonic.

## Technical Validation

### Aggregate eddy verification

**Figure 3:**
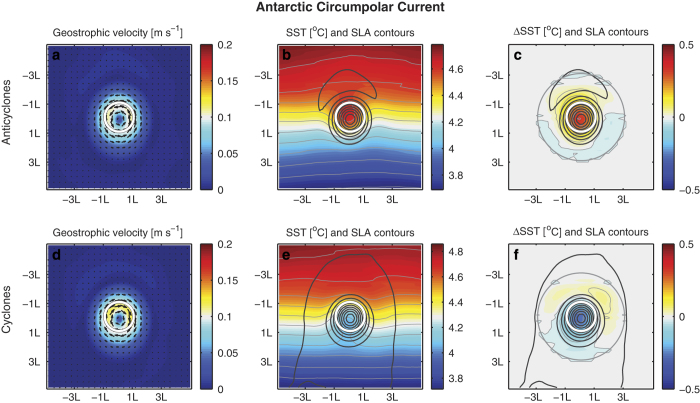
The mean of various variables within the eddy’s vicinity from Antarctic Circumpolar Current eddies. Top panels (**a**–**c**) anticyclonic eddies. Bottom panels d-f: cyclonic. a,d: geostrophic current velocity (colors and arrows). (**b**, **e**) SST (colors) and SLA contours (black solid, 1 cm spacing). (**c**, **f**) SST anomalies (colors) and SLA contours; SST anomalies are relative to the background SST field as shown in **b**, **e** where the background (large scale SST gradient) was interpolated in the eddy impact area (gray circle) from the surrounding SST field. White circle denotes the eddy edge as detected with the algorithm presented here. The x and y axes are given in distance from the eddy center in multiples of eddy radii (L).

One source of difficulty in objectively evaluating eddy identification algorithms is the lack of a precise definition of what constitutes a mesocale eddy in the SLA field^11,32,54^. One way to evaluate the quality of features identified by an algorithm is to observe the aggregate signature such features leave in various fields to see if they are consistent with existing knowledge. Although we do not expect every feature identified to be a real eddy, especially given the noise in the AVISO data, we would expect that cumulatively these features would display known eddy signatures in various fields such as surface velocity or sea surface temperatures. Fig. 3 shows the mean SLA, geostrophic current speed, and SST for *all* (no filtering, such as based on lifespan or amplitude) eddies identified in the Antarctic Circumpolar Current (here defined as the latitude band between 45S°S and 60S°S). The average detected eddy is clearly associated with a ring of intensified geostrophic current speed and current vectors show a rotating feature. Both SLA and current speeds are derived from the same satellite sensors. An independent data source, satellite microwave SST (from http://www.remss.com/) show a perturbation in the large-scale north-south SST gradient (Fig. 3b,e). The SST anomaly is consistent with the known imprint eddies leave on the SST field^21^ (Fig. 3c,f; here the SST anomalies are relative to a background SST field obtained by interpolating b, c in the area denoted by a gray circle in c, f). This confirms that, on average, the features we detect exhibit the spatio-temporal patterns expected from mesoscale ocean eddies (see also Supplementary Information). This analysis, however, is insufficient to say anything about the trapping ability of such features. While ocean eddies are thought to have the potential to trap fluid, it is yet an open question to which extent they do so, with indications that only a few eddies retain their initial waters over long periods^63^. This should be kept in mind when using this dataset for applications, such as estimating heat transport associated with eddies.

### Manual verification

**Figure 4:**
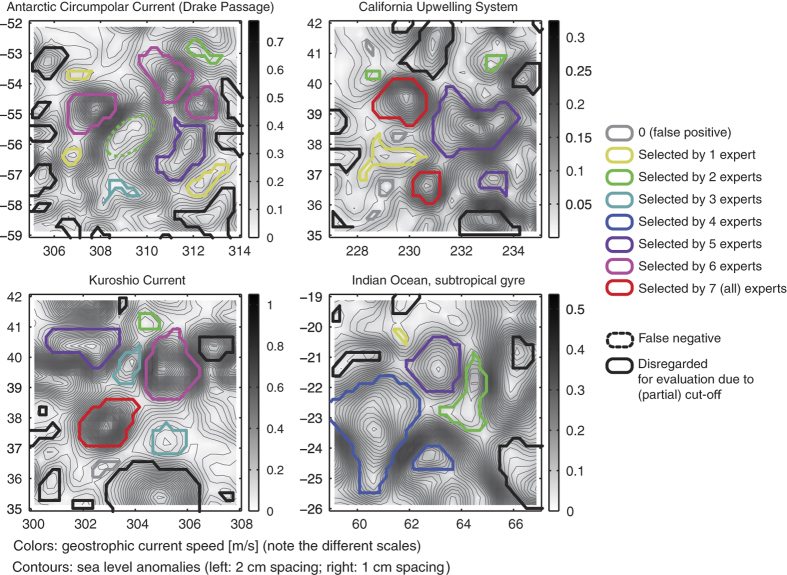
The contours of mesoscale features identified by our algorithm and domain experts.

To assess the algorithm’s ability to extract mesoscale features, we carry out a manual evaluation following the approach of Chaigneau *et al*. (2008)^51^: seven experts (oceanographers) were provided with a snapshot of SLA and geostrophic current speeds in four different regions. The experts marked features which they thought were mesoscale eddies. In total there were 33 mesoscale features across the four regions, of which 27 were identified by both the algorithm and the experts. Five small eddies were only identified by the algorithm. Using these quantities we can calculate the success of detection rate (SDR) and excess of detection rate (EDR)^51^ such that:

$$SDR=\frac{N_{c}}{N_{e}},$$

$$EDR=\frac{N_{om}}{N_{e}},$$

where *N*_*c*_ denotes the number of common eddies identified by at least a single expert and the automated method, *N*_*e*_ corresponds to the total number of eddies identified by the experts, and *N*_*om*_ is the total number of eddies identified only by the automated method. Using these definition our algorithm has a success discovery rate of $\frac{27}{28}=96.4\%$ and an excess discovery rate of $\frac{5}{28}=17.8\%$. However, the expert evaluation was highly variable with only two features identified by all experts. Fig. 4 shows the eddies identified by our method along with the number of experts that identified each feature. The first observation is that, based on a single SLA snapshot, the experts did not agree on what constitutes an eddy. For example, although the algorithm did identify several smaller eddies that the experts did not highlight, there were small sized eddies identified by both the algorithm and the experts (e.g., in the upper left corner in the California Current upwelling system and the Indian Ocean). This supports the inclusive approach we take here, providing users with all features that are potential eddies, and leave it to the user to filter the features. For general purpose studies, we provide some suggestions on how to filter the detected features in the Usage Notes.

**Figure 5:**
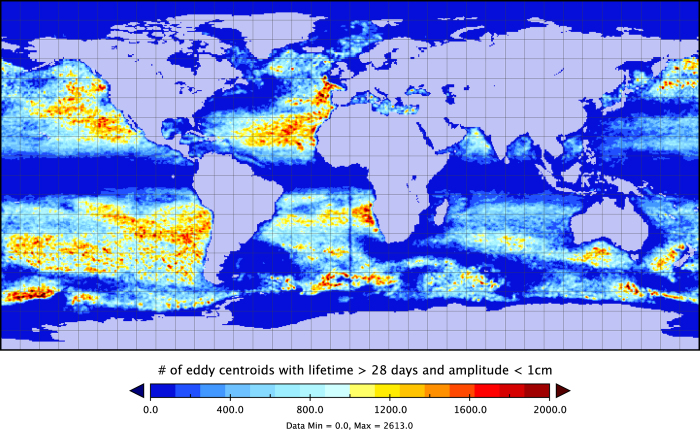
Eddy centroid count for eddies that persisted for more than 28 days yet had a least a single time step with less then one centimeter amplitude. The data are binned in 1×1 degree cells for the 1993–2014 period.

Another observation is that the algorithm simply searches for closed SLA contours which contain a single extrema. In contrast, for the manual detection, the experts took into account additional information, such as the amplitude, the SLA gradient or current speed, the separation of a feature from the larger-scale front, or the shape of a feature. By constraining the algorithm, for instance, to eddies of larger areas/pixel sizes or amplitudes, the algorithm can easily be pushed towards a smaller number of detected eddies. Again, we argue to be careful with such arbitrary thresholds as we find that long-lived eddies can have small amplitudes (Fig. 5). We suggest in the Usage Notes to rather filter by lifespan. This agrees with comments by the eddy detection experts that information about the temporal evolution of the SLA would have been very helpful to distinguish between spurious and real features. This was indeed the case in the only instance where experts identified an eddy while the algorithm did not (eddy with dashed green contour in the Antarctic Circumpolar Current panel). Although our method did not detect the eddy at that time, it did identify the feature as two small eddies in the following time step.

To demonstrate the effect thresholds may have on perceived eddy dynamics, we highlight eddies that persist for more than 28 days yet had amplitudes less than one centimeter in at least one time-step (Fig. 5). Notice the geographic regions where such long-lived eddies with low amplitude cluster. Such regions would be artificially underrepresented if eddies with less than 1 cm amplitude were omitted.

### Verification of our methods on model SLA

We also tested our algorithm’s performance at resolving mesoscale eddies in global climate simulation data. While this evaluation does not directly involve the data presented here, it does give a sense of its quality assuming that SLA observation from satellite altimeters are noisy realizations of SLA from model output. The additional value of a model simulation is that additional physical information is available, such as vorticity, which is independent of the SLA data.

We ran our eddy identification algorithm on daily sea level anomaly (SLA) output from the GFDL CM2.5 High-Resolution Coupled Climate Model^68^. We also calculated daily vorticity from model-output surface current velocities. Overall, we found that the rotational direction of the features detected is consistent with the sign of the vorticity (positive for anticyclonic and negative for cyclonic in the Southern Hemisphere).

The global climate model features a resolution of the ocean of 28 km at the tropics to 8 km at high latitudes (nominally ~25°). We used 180 days of the year 156 of a simulation with a radiative forcing and atmospheric composition of the year 1990 (see Delworth *et al.* (2012)^68^, ‘CM2.5 1990 Control’). We do not expect the results of the validation to be dependent on the chosen simulation or time period.

### Evaluation

**Figure 6:**
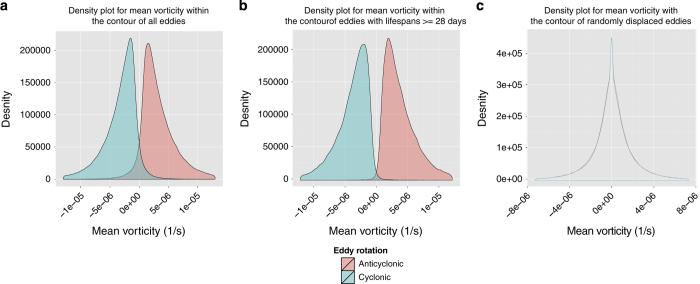
The distribution of mean vorticity within the contour of the eddies identified in model-output SLA in a subsection of the Southern Hemisphere. Panel (**a**): the vorticity distribution for all eddies. (**b**): Same as (**a**) but excluding eddies that did not persist for 28 days. The misclassification rate is the area where the two distributions overlap. (**c**): The distribution of mean vorticity within the contour of eddies after they were randomly displaced from their original location.

Figure 6 (panel a) shows the mean vorticity within the 972,148 eddy contours identified. The majority of eddies identified have the proper vorticity sign with the exception of 6.3% of the features. However, if we remove uncertain features that did not persist beyond 28 days only 1.2% did not have the proper vorticity sign.

To compare the significance of such accuracy, we conducted a randomization experiment to test how likely was the separation between cyclonic and anticyclonic mean vorticity compared to random chance. To do so, for each feature identified in SLA, we randomly moved its location between 0° and 2.5° in either direction (north-south or east-west). Fig. 6 (panel c) shows the distribution of mean vorticity for the eddies when placed in random locations. The cyclonic and anticyclonic mean vorticity distribution are not separated as in Fig. 6 panels (a) and (b), thus showing that our algorithm identifies physically meaningful features from the SLA filed.

**Figure 7:**
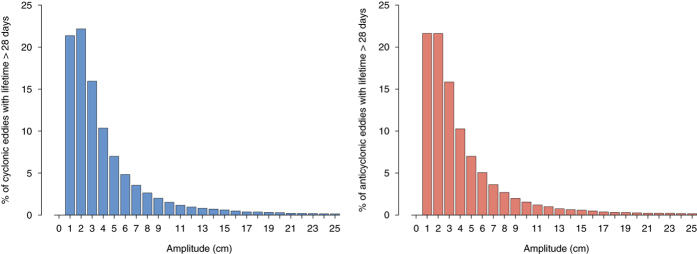
Distributions of the percentage of eddies with amplitude less than a certain value in model output SLA. We selected the features with the proper vorticity sign and kept only those that persisted for more than 28 days. Cyclonic eddies are on the left (blue) while anticyclonic on the right (red). Each bar shows the percentage of eddies that persisted for more than 28 days, had the proper vorticity sign, and had an amplitude less than the value on the x-axis. Notice that 22% of these eddies have an amplitude less than one centimeter (first bar).

The impact of strict thresholds can also be seen in this experiment. In Fig. 7 we show the percentage of eddies from the model data that lived for at least 28 days and had the proper vorticity sign while having amplitudes less than a certain threshold. Thus, the bar on the x-axis at value 2 denotes the percentage of eddies that had an amplitude strictly less than two centimeters. Fig. 7 highlights the impact of imposing thresholds on the physical characteristics of eddies, as 22% of all eddies that persisted for more than 28 days with the proposed vorticity sign had an amplitude less than one centimeter in at least a single time-step. Hence, excluding such features because of failure to pass the commonly-used one centimeter threshold would severely distort reported eddy dynamics especially in regions likely to contain such features as seen in Fig. 5.

### Analysis of eddies with inconsistent vorticity

**Figure 8:**
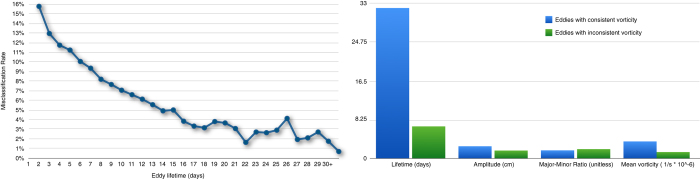
A closer look at the eddies with inconsistent vorticity in model data. Left panel: Misclassification rate (number of eddies with the wrong vorticity/number of eddies found) as a function of eddy lifetime. Shorter lived eddies tend to contribute to the misclassification rate more than longer lived eddies. Right panel: Summary of the difference in means between the eddies with proper vorticity sign (N=911,593) and the misclassified eddies (N=59,839). Standard errors are reported between parentheses. The mean lifetime of proper eddies was 31.99 days (0.04) and 6.88 days (0.04) for misclassified eddies. The mean amplitude was 2.66 cm (0.004) for the properly identified eddies and 1.75 cm (0.009) for misclassified eddies. The mean ratio of major to minor axis was 1.8 (0.00006) for the properly classified eddies and 2.05 (0.003) for misclassified eddies. The mean absolute vorticity within the contour of consistent eddies is 4.51 1/s×10^−6^(7.2×10^−12^), while 1.75 1/s×10^−6^ (1.03×10^−10^) for inconsistent eddies.

We further analyzed the eddies that had the wrong vorticity sign which we define as misclassified in this experiment. We find that the misclassification rate is inversely proportional to the eddy’s lifetime. Short-lived eddies tend to have a significantly higher misclassification rate than longer ones. Fig. 8 (Left panel) shows the misclassification rate based on the lifetime of the eddy. Most of the short-lived eddies have very poor accuracy with 1-day eddies having 16% misclassification rate. In contrast, longer-lived eddies (e.g., 30+ days) have a misclassification rate of 0.7%.

Finally, we also analyze the lifetime, amplitude, deformation ratio, and mean vorticity of the inconsistent eddies relative to those with consistent vorticity. On average, the properly classified eddies had a mean lifetime of 32 days while the misclassified eddies had a mean lifetime of 6.88 days. Furthermore, 10% of the properly classified eddies persisted for a single day versus 30% for the misclassified eddies. Additionally, 31.7% of the features lived less than 7 days for the properly classified eddies, while 71.7% for misclassified. This trend is also apparent in all other variables analyzed and is summarized in Fig. 8 (Right panel). Overall, the misclassified eddies tend to live shorter, have lower amplitudes, be more distorted, and have lower vorticity than the eddies with the proper vorticity sign.

We should also note that our algorithm could miss real eddies (known as false negatives) in the event: (i) the eddy does not have sufficient impact on the sea surface to create a closed-contour anomaly; (ii) we failed to detect an extremum because two values in the 5×5 neighborhood have the same exact extreme value (e.g., two grid cells tie for the maximum value in the neighborhood)—however this never happened in the AVISO dataset; (iii) if two extrema are so close in space that neither extremum is big enough to reach four grid-cells in size without incorporating both extrema within its contour.

## Usage Notes

### Running the eddy identification software in parallel

One of the advantages of our software is that it was designed to be highly parallel—it can be run simultaneously on multiple independent SLA snapshots to reduce computational time by orders of magnitude. However, our eddy identification software is written in the MATLAB programming language. Certain features of our software require for-pay MATLAB libraries. Unfortunately, the number of MATLAB toolbox licenses available limit the number of parallel instances that can be run simultaneously, as each parallel instance will require its own MATLAB license. To circumvent this limitation we provide a ‘compiled’ version of our software that can be run in parallel without the need of multiple MATLAB toolbox licenses.

We advise users to study our source code first, and then use the compiled version for efficiency. We also provide the source code of the script that we compiled should you want to modify the methods we provide. To compile the source code simply run: ‘mcc -m eddyscan_compiled_script.m’ as a command in MATLAB. Please note that to compile MATLAB code you must have a valid license for the MATLAB Production Server. We do not require you to compile your own version of our eddyscan code, as we have provided all the compiled files on our repository. To run the compiled code you need a copy of the MATLAB Compiler Runtime (MCR) version 8.3 (R2014a) which is free to download and install, and is hosted at http://www.mathworks.com/products/compiler/mcr/ Note: version 8.3 is required for the proper execution of the compiled code, as this is the R2014a version of MCR, and the code was compiled by MATLAB’s R2014a version.

Our compiled source code is located in our repository at ‘OceanEddies/compiled_code/’. To run the MATLAB compiled code on a Unix-based operating system, run the following command:

‘./run_eddyscan_compiled_script.sh <Your-MCR-Directory-Here> <year/sla-daily-data-file.nc> <path-to-sla-directory> <path-to-save-directory>’

<Your-MCR-Directory-Here> is the path to your MCR directory.

<year/ssh-daily-data-file.nc> has two parts. <year> is the year that the SLA file belongs to, e.g., 2004, 2005, etc. <year> is part of this argument because AVISO data is stored in year sub-directories. <sla-daily-data-file.nc> is the NetCDF SSH file.

<path-to-eddy-directory> is the path to your main SLA directory. This is the parent directory of the year sub-directories.

<path-to-save-directory> is the path to the directory where you want to save the resulting detected eddies.

Reference the README file located in our repository at ‘OceanEddies/compiled_code/’ for more detailed information.

### Viewing eddy trajectories

We provide an open-source eddy trajectory viewer in the MATLAB programming language to visualize eddy trajectories (including those provided by CSS11). The viewer is hosted in the software repository along with the eddy identification software under the ‘tracks_viewer’ folder. It is possible to visualize your own eddy trajectories by saving them to the ‘tracks_viewer/track_data/’ folder. We have included a sample dataset in that folder as an example.

There are two ways to set up the tracks viewer. Data can be set up manually, with the steps to do so covered later in this section, or we provide a function called *complete_run.m* that will take care of every step of the process, from running the identification algorithm on SLA data all the way to automatically formatting eddy and track data and storing them in the appropriate location for the viewer to use.

The other alternative to preparing data for the tracks viewer is to format the data yourself, with the help of a function called *prepare_eddy_data_for_viewer.m*, which can be found in the repository at ‘OceanEddies/track_lnn/’. This function will appropriately format data to be used in the viewer. You will then need to manually move the resulting trajectories and associated background SLA to the appropriate folders. We provide an example of where all the data should in ‘OceanEddies/tracks_viewer/’.

### Accessing individual eddy data from eddy trajectories

One common use of the software is to analyze eddies at a trajectory level and then at an individual feature level. For instance, one might be interested in analyzing all trajectories that last at least 30 days and propagate eastward. Once those trajectories have been identified, there might be an interest in analyzing specific characteristics of such trajectories such as size or amplitude. In the trajectory data, each row represents an eddy at a particular time. The third (date index) and fourth (eddy id) columns are needed to uniquely identify an eddy ‘object’ from the database. First, pass the value in the third column to the dates file (‘dates.mat’). This returns an eight-digit date value for the day of the observation (yyyymmdd). Anticyclonic tracks and cyclonic tracks are stored as separate files, so the rotation of the eddy should be known by the file name. Now you may load the daily eddy file using the rotational direction and the data (e.g., ‘cyclonic_20100901.mat’). Loading the corresponding file will open all cyclonic eddy features identified on that date. To access the exact eddy of interest, simply use the unique eddy id from the fourth column in the trajectory data (e.g., ‘eddies20’).

Trajectory data is also available in a Chelton-like format (similar to the data from: http://cioss.coas.oregonstate.edu/eddies/nc_data.html). If the function *complete_run.m* is used to generate tracks, this Chelton-like track format will be automatically generated and saved inside the specified tracks_save_path (path specified as a parameter to complete_run). If complete_run is not used, a Chelton-like track format can be generated by running the function *reformat_track_data_to_chelton.m* with the appropriate parameters. This function can be found in the repository at ‘OceanEddies/track_lnn/’.

### Interpretation of the dataset

Two separate sources of uncertainty are important to consider when using the eddy dataset: the first arises from errors in the input data (AVISO), the second from uncertainties associated with the detection and tracking algorithms which we discussed in the Methods and Validation sections.

The potential of AVISO data to resolve mesoscale features depends on both, the number of satellites capturing altimetry measurements and the postprocessing steps performed to construct the continuous SLA field from the raw data. Major postprocessing steps affecting the mesoscale signal in SLA are the smoothing of the along-track data and the mapping procedure which involves a covariance function of certain spatial and temporal scales (see Chelton *et al.* (2003)^69^, Appendix A of CSS11, and the SSALTO/DUACS User Handbook and AVISO website). Based on a spectral analysis of a previous AVISO version, CSS11 concluded features were resolved sufficiently if of temporal scales around a month and of spatial e-folding scales of approximately 0.4°, corresponding to a diameter of several tens to roughly 100 km, depending on the latitude (features at this scale are still attenuated by a factor of about two (CSS11); an amplitude unattenuated by the mapping procedure is found at scales of 0.6° and larger.) As a result, oceanographers tend to limit their analysis to features that persist over a month and span at least 8 pixels.

The AVISO data used in the present study is different from the data used by CSS11 in two respects. First, our study uses the AVISO SLA incorporating all available satellite sensors that is now labeled ‘all sat merged’ (previously ‘upd’), in contrast to the ‘ref’ data used by CSS11 that includes two sensors only. Second, the most recent version of AVISO was used (released in spring 2014) with a refined postprocessing procedure. Both of these points lead to a better resolution of mesoscale features. Consequently, a better resolution capacity than 8 pixels may be expected. We therefore relax the constraint on minimum eddy size because of the potentially higher fidelity of the AVISO data as well as the assumption that numerous eddies may be small at the beginning and ends of their lifetimes. Hence, we suggest to use lifetime as a filtering criteria and suggest a minimum lifetime of at least 28 to 30 days. For studies targeting a certain type of features (e.g., short-lived, small amplitude, etc.) or region, we provide the entire unadulterated dataset of all mesoscale features that can be analyzed based on the investigators’ discretion.

## Additional Information

**How to cite this article**: Faghmous, J. H. *et al.* A daily global mesoscale ocean eddy dataset from satellite altimetry. *Sci. Data* 2:150028 doi: 10.1038/sdata.2015.28 (2015).

## Supplementary Material



Supplementary Table 1

Supplementary Information

## Figures and Tables

**Table 1:** A comparison between the features in this data descriptor [Data Citation 1] and the data published by CSS11 (ref. 33).

	**Faghmous** ***et al.***	**Chelton** ***et al.***
Spatial resolution	0.25	0.25
Temporal resolution	Daily and weekly	weekly
Temporal coverage	1993–2014	1992–2012
Track length	1 day or more	4 weeks or more
Minimal Size	4+ pixels	8+ pixels
Minimal amplitude	none	1 cm
Exact eddy contour	yes	no
Robust to artificial merges	yes	no
Robust to ‘missing’ eddies	yes	no

**Table 2:** Sample eddy trajectory data. See the ‘Data Records’ section for description of each column.

**Track ID**	**Latitude**	**Longitude**	**Time ID**	**Eddy ID**	**Flag**	**Cyc**
1	−66.227	−108.80	1	109	0	1
1	−66.352	−109.06	2	219	0	1
2	−43.251	83.21	1	897	0	1
2	−41.294	81.08	2	910	0	1
2	−40.843	80.34	3	413	1	1
...	...	...	...	...	...	...
40	45.312	−88.17	40	78	0	1

## References

[d1] Dryad Faghmous J. H. 2015 http://dx.doi.org/10.5061/dryad.gp40h

