# Supplementary material for: A daily global mesoscale ocean eddy dataset from satellite altimetry
Source: Sci Data. 2015 Jun 9;2:150028. doi: 10.1038/sdata.2015.28 (PMC4460914; doi:10.1038/sdata.2015.28)
Supplement: Supplementary Information [file sdata201528-s3.pdf]

Supplementary Materials

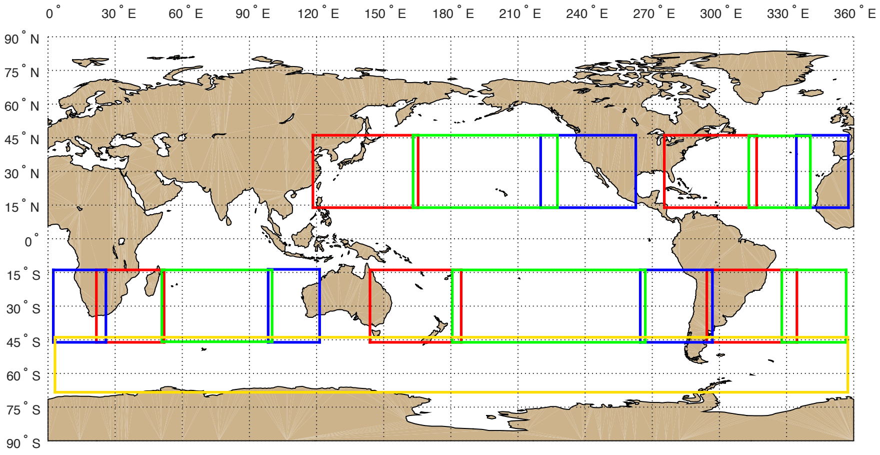

Figure S1. Four regions were we compute eddy composites (figures S2-S7). The four regions are: Antarctic Circumpolar Current, yellow rectangle (figure 4); Eastern boundary regions, blue rectangles; Western boundary regions, red rectangles; and subtropical gyres, green rectangles.

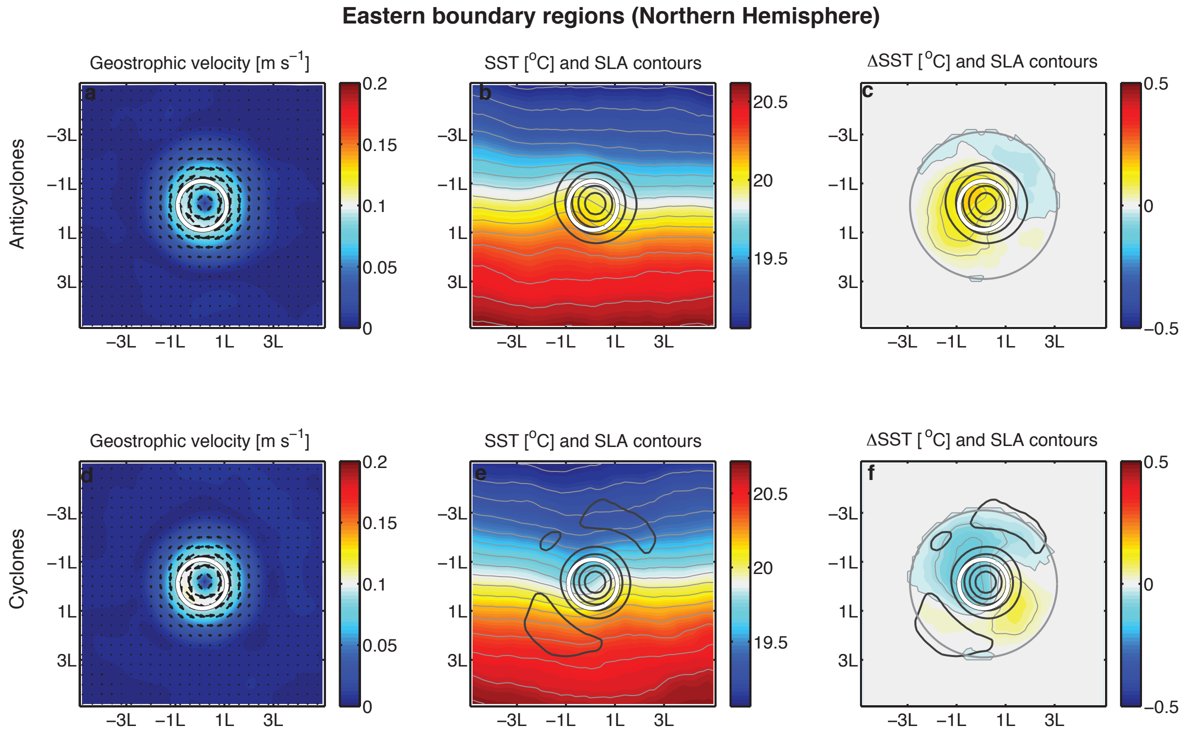

Figure S2. The mean of various variables within the eddy's vicinity from eastern boundary (blue rectangles in figure S1) eddies in the Northern Hemisphere. Top panels a to c: anticyclonic eddies. Bottom panels d to f: cyclonic. a,d: geostrophic current velocity (colors and arrows). b, e: SST (colors) and SLA contours (black solid, 1 cm spacing). c, f: SST anomalies (colors) and SLA contours; SST anomalies are relative to the background SST field as shown in b, e where the background (large scale SST gradient) was interpolated in the eddy impact area (gray circle) from the surrounding SST field. White circle denotes the eddy edge as detected with the algorithm presented here. The x and y axes are given in distance from the eddy center in multiples of eddy radii (L).

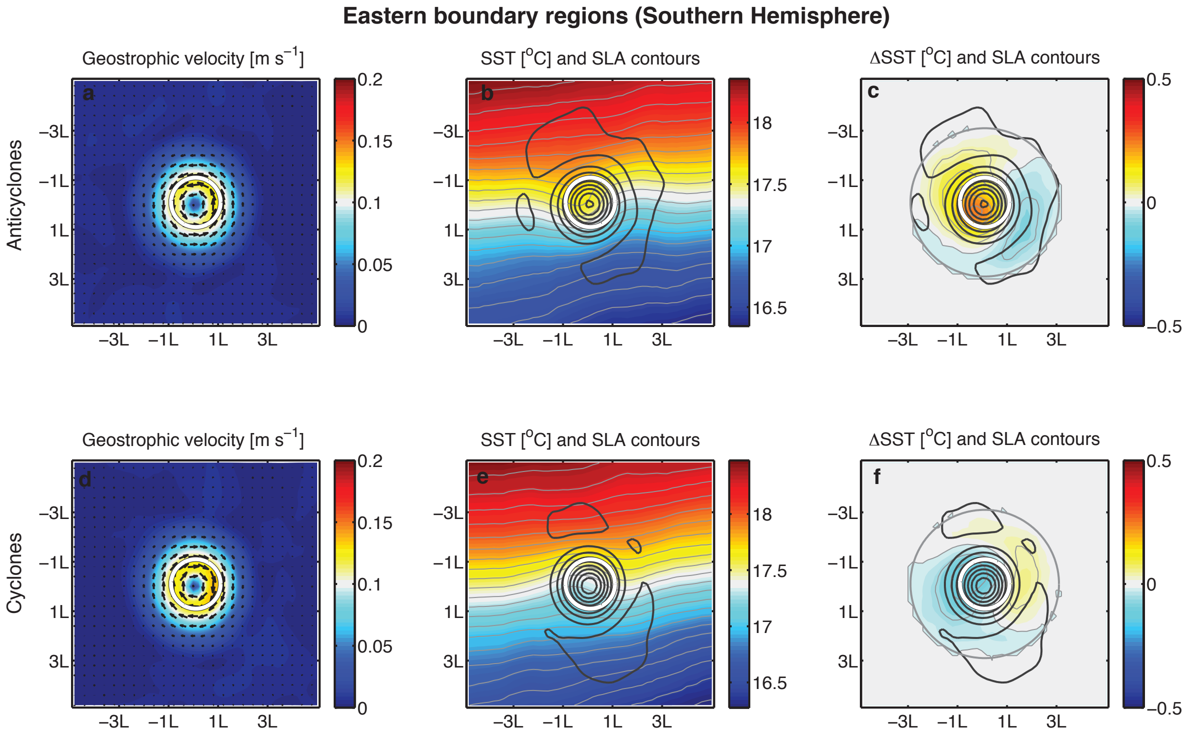

Figure S3. As in figure S2 except for eastern boundary (blue rectangles in figure S1) eddies in the Southern Hemisphere.

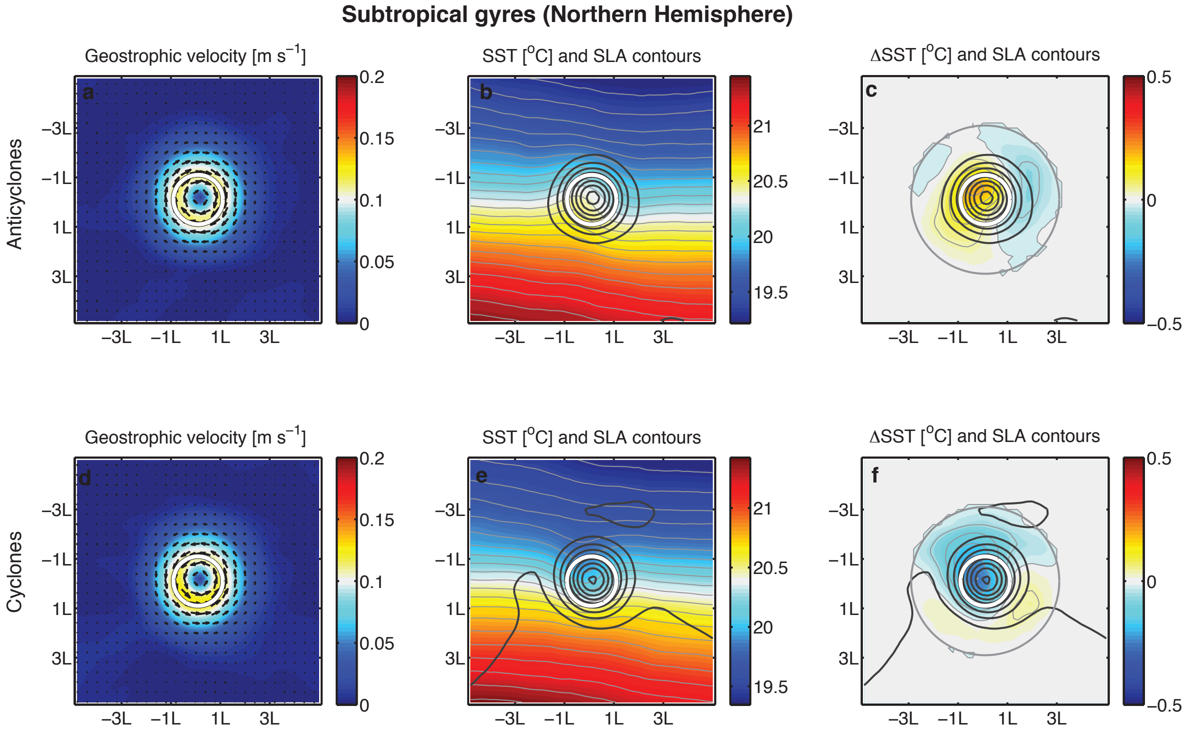

Figure S4. As in figure S2 except for subtropical gyres (green rectangles in figure S1) eddies in the Northern Hemisphere.

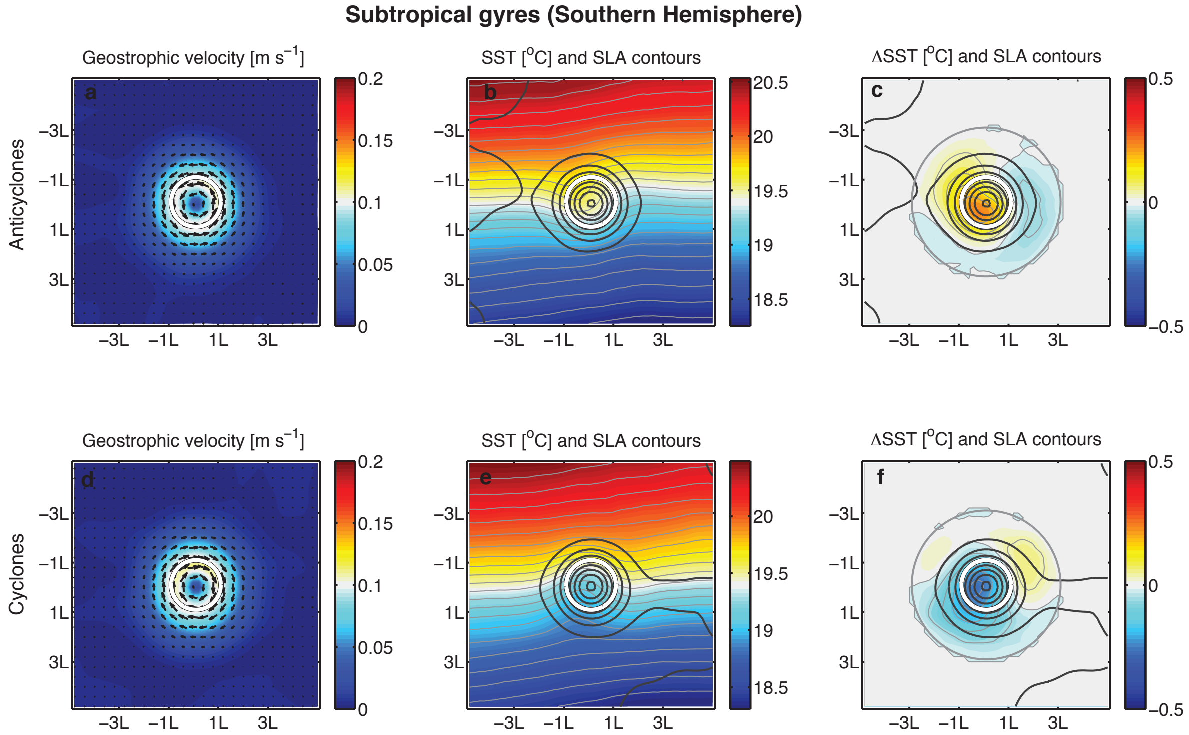

Figure S5. As in figure S2 except for subtropical gyres (green rectangles in figure S1) eddies in the Southern Hemisphere.

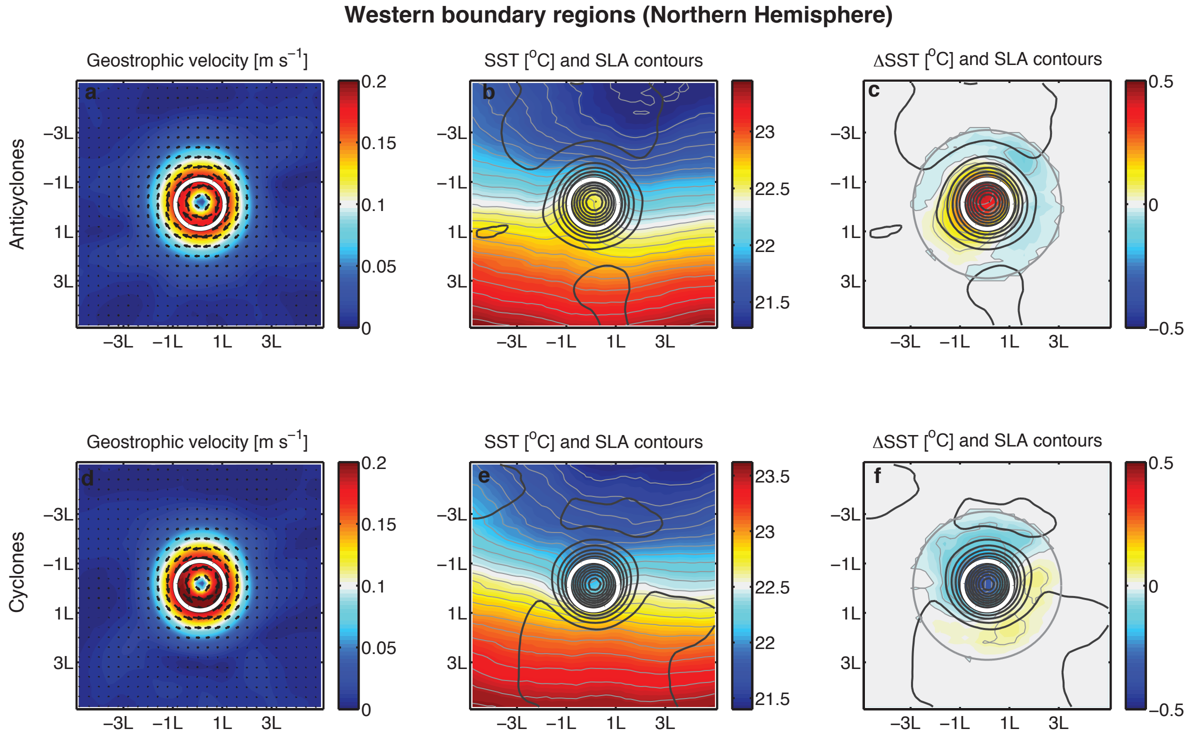

Figure S6. As in figure S2 except for western boundary (red rectangles in figure S1) eddies in the Northern Hemisphere.

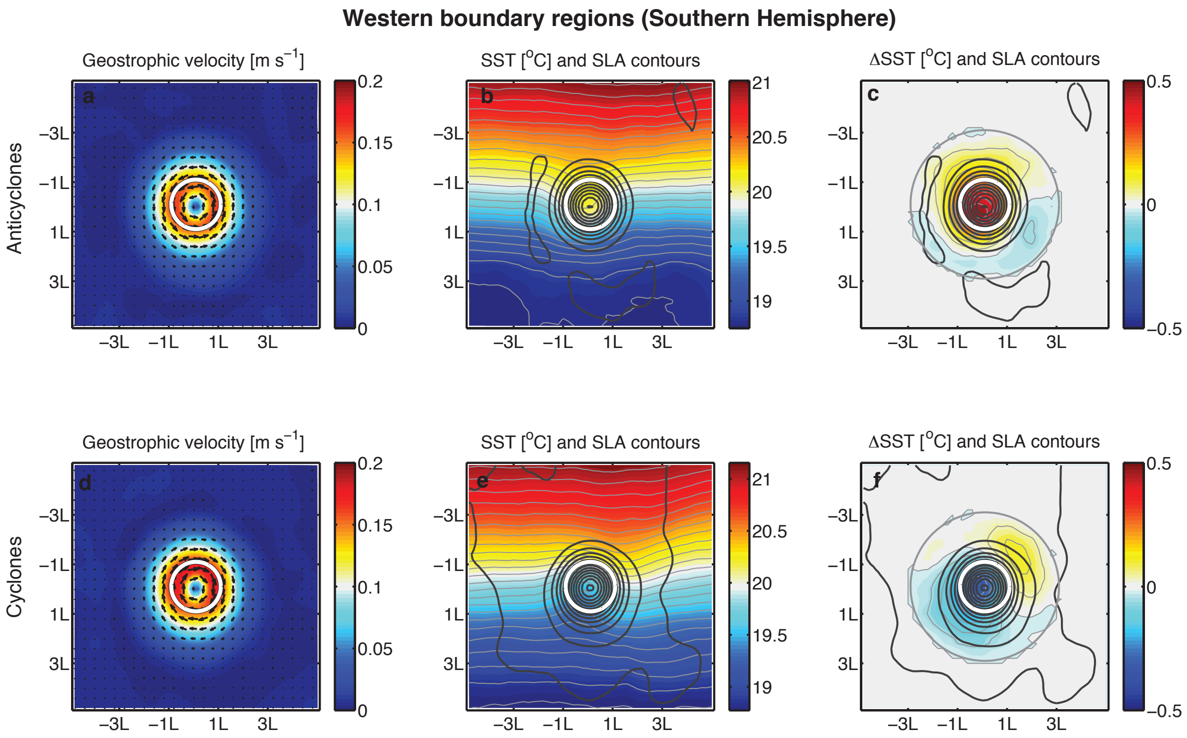

Figure S7. As in figure S2 except for western boundary (red rectangles in figure S1) eddies in the Southern Hemisphere.
